# Sequencing of *BRCA1/2*-alterations using NGS-based technology: annotation as a challenge

**DOI:** 10.18632/oncotarget.28213

**Published:** 2022-03-03

**Authors:** Silvana Ebner, Ria Winkelmann, Saskia Martin, Jens Köllermann, Peter J. Wild, Melanie Demes

**Affiliations:** ^1^Dr. Senckenberg Institute of Pathology, University Hospital Frankfurt, Frankfurt am Main, Germany; ^2^Wildlab, University Hospital MVZ GmbH, Frankfurt am Main, Germany

**Keywords:** *BRCA1/2*, annotation, degree of pathogenicity, next-generation sequencing, NGS

## Abstract

In this study, the molecular profile of different *BRCA*-associated tumor types was assessed with regard to the classification and annotation of detected *BRCA1/2* variants. The aim was to establish guidelines in order to facilitate the interpretation of *BRCA1/2* alterations in routine diagnostics. Annotation of detected variants was evaluated compared to background mutations found in normal tissue samples and manually reviewed according to distinct online databases. This retrospective study included 48 samples (45 tumors, three non-tumors), which were sequenced with the GeneReader (QIAGEN). Thereof ten samples were additionally analyzed with the Ion S5™ (Thermo Fisher) and 20 samples with the MiSeq™ (Illumina^®^) to compare the different NGS devices, as well as the sequencing results and their quality. The analysis showed that the individual NGS platforms detected different numbers of *BRCA1/2* alterations in the respective tumor sample. In addition, the GeneReader revealed variability in the detection and classification of pathogenic alterations within the platform itself as well as in comparison with the other platforms or online databases. The study concluded that the Ion S5™ in combination with the Oncomine™ Comprehensive Assay v3 is most recommendable for current and prospective requirements of molecular analysis in routine diagnostics. In addition to the two *BRCA1/2* genes, a broad number of other genes (*BRCA*ness genes and genes involved in the repair pathway) is covered by the panel, which may open up new treatment options for patients depending on the respective eligibility criteria.

## INTRODUCTION

Germline mutations in *BRCA1/2* cause the hereditary breast and ovarian cancer (HBOC) syndrome, an autosomal dominant disease [1–3], that lead to an increased lifetime risk of developing these cancers, and a risk for the development of a broad range of other cancer types (i.e., prostate or pancreatic cancers) [4]. Mutations in *BRCA1/2* are predicted to disrupt protein-protein interactions, necessary for DNA repair [5–8]. Both proteins have distinct functional domains. In BRCA1 the RING finger is a protein-protein interaction domain which may have ubiquitin ligase activity. Another protein-protein interaction motif is the BRCT domain that is found predominantly in proteins involved in DNA repair [9]. BRCA2 contains nine separate potential RAD51 binding domains: eight BRC repeats and a structurally independent carboxy-terminal domain. Both BRCA1 and BRCA2 contain nuclear localization sequences (NLS) [9]. While BRCA1 functions as a pleiotropic DNA damage response protein in both checkpoint activation and DNA repair, BRCA2 is a mediator of the core mechanism of homologous recombination [10, 11]. Therefore BRCA1 and BRCA2 are involved in regulating cellular processes including transcription, cell cycle regulation and DNA damage response, with a particularly important role in DNA repair during homologous recombination (HR) [10]. BRCA1/2 form protein complexes with known tumor suppressors including RAD51, BARD1 and PALB2. Especially, BRCA1 and BARD1 facilitate resection of DNA ends and enhance the activity of the recombinase RAD51 [12, 13], whereas BRCA2 binds single-stranded DNA and loads RAD51 monomers at sites of DNA double-strand breaks [12]. Since BRCA1 and BRCA2 interact in such a large protein complex, next-generation sequencing (NGS) approaches are recommended for a mutational analysis that also include *BRCA* associated genes (so-called *BRCA*ness genes: *ATM, CHEK2, CDK12, FANCA, NBN*, *PALB2, RAD51,* etc.,) or other tumor suppressor genes such as *TP53* and *PTEN*.

Genomic variants in *BRCA1/2* can be subdivided into three classes: single nucleotide variants (SNVs), small insertion or deletion events (indels) and large genomic rearrangements (LGRs). A valid classification according to the degree of pathogenicity (benign, pathogenic or variance of unknown significance (VUS) is essential for therapeutic indication. Pathogenic *BRCA1/2* SNVs and indels can be found widely distributed throughout the coding sequence and conserved intronic sequences of both genes, which means that there are no hotspot mutations [14]. Therefore, for the analysis of *BRCA1/2* alterations it is also necessary to sequence the entire coding region as well as exon/intron junctions. Since the genes are very large, sequencing results in a plethora of reads that must be carefully evaluated. Furthermore, BRCA1 has an unusual high density of *Alu* DNA sequences (repetitive DNA sequences) [15] and BRCA2 contains repetitive sequences that act as RAD51 binding domains [9]. Both can cause erroneous read alignment and consequently artificial detection of mutations. Because of their size, LGRs cannot be detected with NGS methods. A cytogenetic analysis is required for this purpose, so possible LGRs are not considered in the present study.

Determining the mutation status of *BRCA1/2* is extremely important for a patient. Typically, a person with a *BRCA1/2* mutation is eligible for treatment with a poly (ADP-ribose) polymerase (PARP) inhibitor [16–20]. The most prominent example is Olaparib. This PARP inhibitor has already been approved by the *European Medicines Agency* (EMA) for breast and ovarian cancer and recently also for *BRCA1/2* mutations in prostate cancer.

Next-generation sequencing analyses are increasingly requested in the context of molecular diagnostics. As the trend increases towards individual and personalized medicine, more and more manufacturers are developing their own sequencing platforms designed for routine diagnostics. NGS is a high-throughput methodology that enables rapid sequencing of many genes in parallel. In this study a collective of distinct tumor samples as well as normal tissue was sequenced with the GeneReader (QIAGEN), as this device was the established sequencing platform and diagnostic standard in our institute at the time of data collection. Additionally, some samples were sequenced with either the Ion S5™ (Thermo Fisher) or the MiSeq™ (Illumina^®^) in order to verify the plausibility of the sequencing results generated by the GeneReader. The specific strategy of each NGS platform differs in terms of sequencing technology, sequencing depth, sensitivity, determination of quality and quantity parameters, bioinformatics as well as the platform’s usefulness for particular applications [21]. The NGS platforms used in this study are amplicon-based strategies, in which genomic regions are selectively captured from a DNA or RNA sample before sequencing [22]. All devices operate via the sequencing-by-synthesis (SBS) method, in which individual bases are incorporated step by step and detected directly. However, while the GeneReader and the MiSeq™ detect fluorescence signals, the Ion S5™ measures a change in the pH value.

Further purpose of this study was to evaluate the reliable clinical applications of next-generation sequencing in terms of laboratory work, expenditure of time, cost efficiency, bioinformatics evaluation and finally the correct annotation of detected *BRCA1/2* alterations correlated with distinct quality criteria. Important quality criteria were the tumor cell content of the FFPE samples and the DNA concentration used for library preparation. For the credibility of detected alterations, additional cut-off values for allele frequency (AF, ≥5%) and coverage (≥100× for GeneReader and ≥500× for Ion S5™ and MiSeq™) were set. Only those variants that met these quality criteria were included in the study. To extend the comparison, three normal tissue samples were analyzed additionally, allowing assumptions about germline mutations, polymorphisms or artefacts. However, a cytogenetic analysis would be necessary in a next step for a more precise assessment and to supplement the NGS data. Another aim was to identify artefacts and to classify variations of uncertain significance (VUS) as well as pathogenic alterations more accurately. The results were used to determine which device is most recommendable for reliable routine diagnostics. Based on the knowledge gained, supporting tools were created to facilitate future NGS evaluations.

## RESULTS

### Panel-specific gene coverage

In order to analyze the sequencing results of the three different NGS platforms used, the gene coverage of the corresponding sequencing panel was investigated and compared to the reference genome sequence. For *BRCA1* the reference sequence NM_007294 was used and for *BRCA2* NM_000059 (available from the National Center for Biotechnology Information at https://www.ncbi.nlm.nih.gov/). The following Table 1 shows a coverage comparison (number of base pairs, bp) of the distinct NGS panels used as well as the respective reference sequence.

**Table 1:** Comparison of *BRCA1* and *BRCA2* gene coverage of the different sequencing panels used

Gene	GeneRead™ QIAact BRCA UMI Panel	Oncomine™ Comprehensive Assay v3	AmpliSeq™ BRCA Panel for Illumina^®^	RefSeq
***BRCA1***	6,463 bp	10,382 bp	10,599 bp	5,589 bp
***BRCA2***	11,236 bp	15,131 bp	15,131 bp	10,270 bp

Total number of base pairs (bp) covered by the amplicons of the distinct sequencing panels and the reference sequences (RefSeq).

### *BRCA1/2* variant calling

Looking at the average number of *BRCA1* and *BRCA2* variants detected in the tumor and non-tumor samples, the GeneReader and the Ion S5™ each found on average 11.5 mutations per sample. In contrast, the MiSeq™ detected on average seven mutations more per sample (18.5). This includes pathogenic and non-pathogenic mutations, that met the quality criteria described previously. The pathogenicity of all *BRCA1* and *BRCA2* variants detected in the tumor samples examined was classified according to the scheme seen in Table 2, which also represents the basis of the annotation of the online database ClinVar [23, 24].

**Table 2:** 5-tier classification system for sequence variants identified by genetic testing (modified [24])

Class	Description	Probability of being pathogenic
**5**	Definitely pathogenic	<0.99
**4**	Likely pathogenic	0.95–0.99
**3**	Uncertain	0.05–0.949
**2**	Likely not pathogenic or of little clinical significance	0.001–0.049
**1**	Not pathogenic or of no clinical significance	<0.001

**Figure 1:**
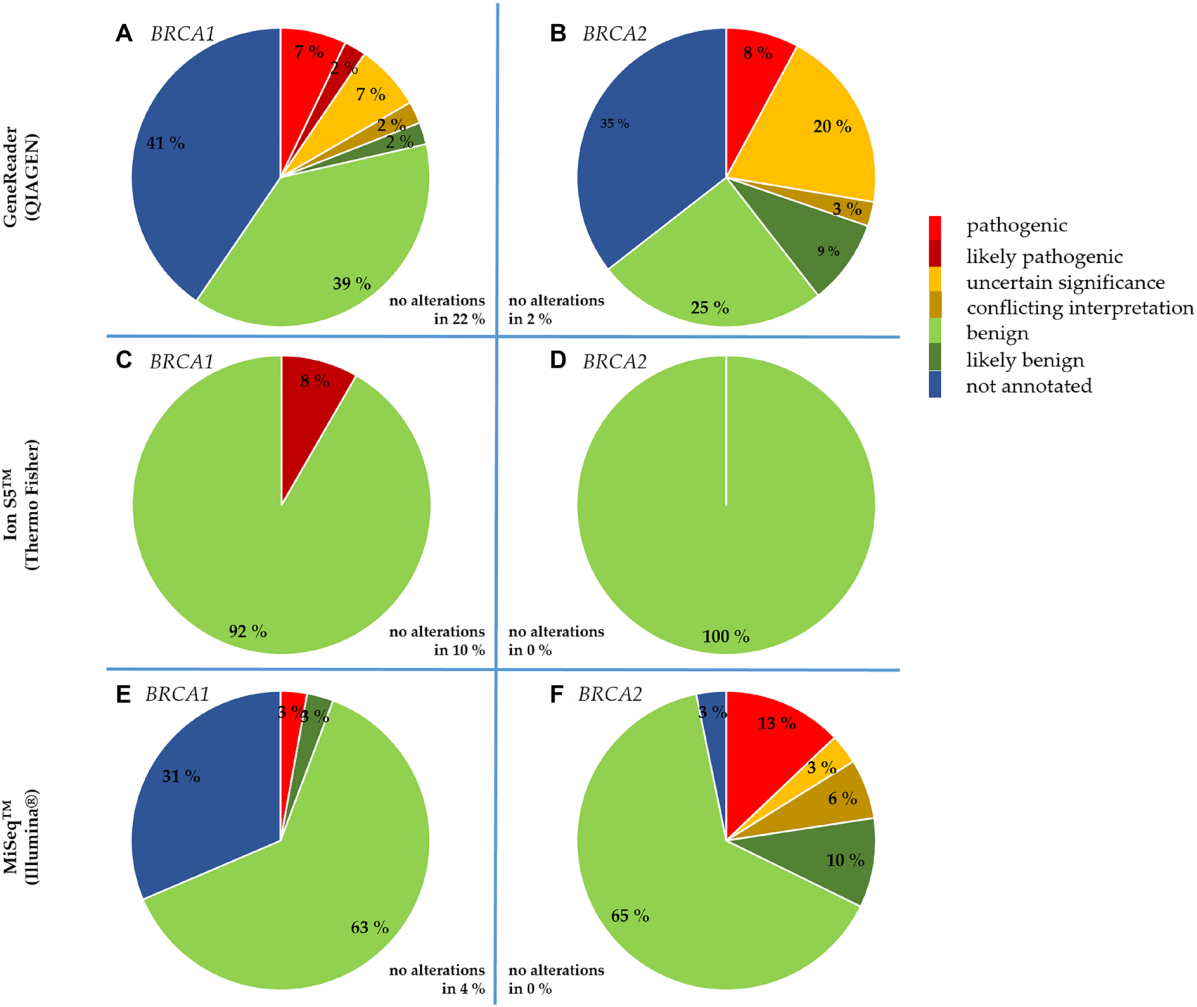
NGS platform-dependent comparison of the clinical significance of *BRCA1* and *BRCA2* alterations detected in the tumor samples. Alterations detected with the GeneReader in *BRCA1* (**A**) and *BRCA2* (**B**). Alterations detected with the Ion S5™ in *BRCA1* (**C**) and *BRCA2* (**D**). Alterations detected with the MiSeq™ in *BRCA1* (**E**) and *BRCA2* (**F**).

A platform-dependent overview of the classification of detected variants is given in Figure 1. Since different numbers of samples were sequenced per platform, all values are given as percentages for better comparison. The variants classified as pathogenic or likely pathogenic were all annotated in ClinVar as “reviewed by expert panel”. No *BRCA1* alterations were found in 22% of the tumor samples sequenced with the GeneReader, in 10% with the Ion S5™ and in 4% with the MiSeq™, whereas no *BRCA2* alterations were detected in 2% of the tumor samples sequenced with the GeneReader, but not with the Ion S5™ or the MiSeq™. Some *BRCA1/2* variants detected with the GeneReader or the MiSeq™ could not be found in any of the databases mentioned, so they were classified as “not annotated”. Those not annotated *BRCA1/2* variants detected with the MiSeq™ were exclusively intronic mutations. In contrast, the variant types of not annotated mutations detected with the GeneReader were mainly missense mutations (*BRCA1*: 82%, *BRCA2*: 76%). Other types were large indel mutations (*BRCA1:* 18%, *BRCA2*: 12%) as well as intronic mutations (*BRCA2*: 12%).

During evaluation of variant calling, three *BRCA1* and nine *BRCA2* alterations could be detected with the GeneReader, that exhibited a pathogenic ClinVar classification. One pathogenic *BRCA1* alteration was detected with the Ion S5™ and also one pathogenic *BRCA1* as well as five pathogenic *BRCA2* alterations were detected with the MiSeq™. In some cases, QIAGEN exhibited inconsistencies in the detection and classification of pathogenic alterations within the platform as well as in comparison with the other platforms or online databases. In this context, variants assessed as pathogenic on ClinVar were classified by QIAGEN as likely pathogenic or even as variants of uncertain significance.

For all NGS platforms a list of benign background mutations could be created as a helping tool (Table 3). This allows the focus of the evaluation to be shifted to pathogenic, therapy-relevant mutations or the closer examination of VUS. The results of all samples were included, whereas variants found in normal tissue were marked with an asterisk (^*^).

**Table 3:** Benign *BRCA1* and *BRCA2* variants detected with the different NGS platforms

	*BRCA1*			*BRCA2*		
	GeneReader	Ion S5™	MiSeq™	GeneReader	Ion S5™	MiSeq™
**Benign SNPs**		c.5152+66G>A	c.5152+66G>A		c.-26G>A	c.-26G>A^*^
			c.5075-53C>T		c.681+56C>T	c.681+56C>T
			c.4956G>A	c.865A>C		c.865A>C
		c.4937-68A>G	c.4937-68A>G	c.1114A>C^*^	c.1114A>C	c.1114A>C^*^
	c.4837A>G^*^	c.4837A>G	c.4837A>G	c.1365A>G	c.1365A>G	c.1365A>G
	c.4308T>C^*^	c.4308T>C	c.4308T>C	c.2229T>C	c.2229T>C	c.2229T>C
	c.3548A>G	c.3548A>G	c.3548A>G	c.2971A>G	c.2971A>G	c.2971A>G
	c.3113A>G	c.3113A>G	c.3113A>G	c.3396A>G	c.3396A>G	c.3396A>G^*^
	c.2612C>T	c.2612C>T	c.2612C>T	c.3807T>C	c.3807T>C	c.3807T>C
	c.2311T>C	c.2311T>C	c.2311T>C	c.4563A>G^*^	c.4563A>G	c.4563A>G^*^
	c.2082C>T	c.2082C>T	c.2082C>T	c.5744C>T	c.5744C>T	
	c.2077G>A		c.2077G>A	c.6513G>C^*^	c.6513G>C	c.6513G>C^*^
	c.1067A>G^*^			c.7242A>G	c.7242A>G	c.7242A>G
		c.442-34C>T	c.442-34C>T	c.7397T>C^*^	c.7397T>C	c.7397T>C^*^
					c.7435+53C>T	c.7435+53C>T
				c.7806-14T>C^*^	c.7806-14T>C	c.7806-14T>C
						c.8755-66T>C
				c.10234A>G		
**Benign SNVs**		c.4987-92A>G	c.4987-92A>G			c.425+67A>C
	c.4535G>T		c.4535G>T			
			c.4358-2885G>A			
	c.2859T>C			c.1395A>C	c.1395A>C	
	c.1866G>C			c.2913A>G		
	c.1648A>C		c.1648A>C	c.4068G>A		c.4068G>A
	c.1533C>G			c.6100C>T		
	c.1456T>C		c.1456T>C	c.6297A>G^*^		
	c.1308T>A^*^			c.7188G>A		
	c.536A>G		c.536A>G	c.7296A>G		
				c.8851G>A	c.8851G>A	c.8851G>A
				c.9976A>T		
				c.10191C>G		c.10191C>G^*^
				c.^*^1G>C		
**Benign Indels,** **Duplications,** **MNVs**			c.4185+21_ 4185+22delTG			c.68-7dup
			c.548-58del			c.793+63del
			c.441+18CTT [6]	c.1909+11_ 1909+12insT		
			c.441+52_ 441+63del			c.1909+22dup

Abbreviations: *BRCA1/2*, SNP: single nucleotide polymorphism; SNV: single nucleotide variant; MNV: multi nucleotide variant. Variants also detected in normal tissue sample marked with an asterisk (^*^).

In most cases these alterations were synonymous single nucleotide variants (SNV) and thus classified as benign. If a variant occurs in at least 1% of the population [25], it is termed as single nucleotide polymorphism (SNP), which is generally assumed to have no clinical relevance. All detected mutations were matched with the database gnomAD, which provides the global allele frequency of certain variants. The detected benign mutations were divided into (1) SNPs, (2) SNVs and (3) indels, duplications and multi nucleotide variants (MNVs).

### Inter-platform concordance of detected *BRCA1/2* variants

Based on the collected data, this study was intended to evaluate sequencing results of the GeneReader. In order to validate these results a comparison with the other NGS platforms (Ion S5™ and MiSeq™) was performed, especially with regard to the concordance of the detected pathogenic and non-pathogenic variants. So, the variant calling results of the samples analyzed with the GeneReader were compared with either the Ion S5™ (*n* = 10) or the MiSeq™ (*n* = 20), as well as the Ion S5™ with the MiSeq™ (*n* = 1). 18 samples were sequenced with the GeneReader only, thus the results of these analyses were not included in the following comparison.

**Figure 2:**
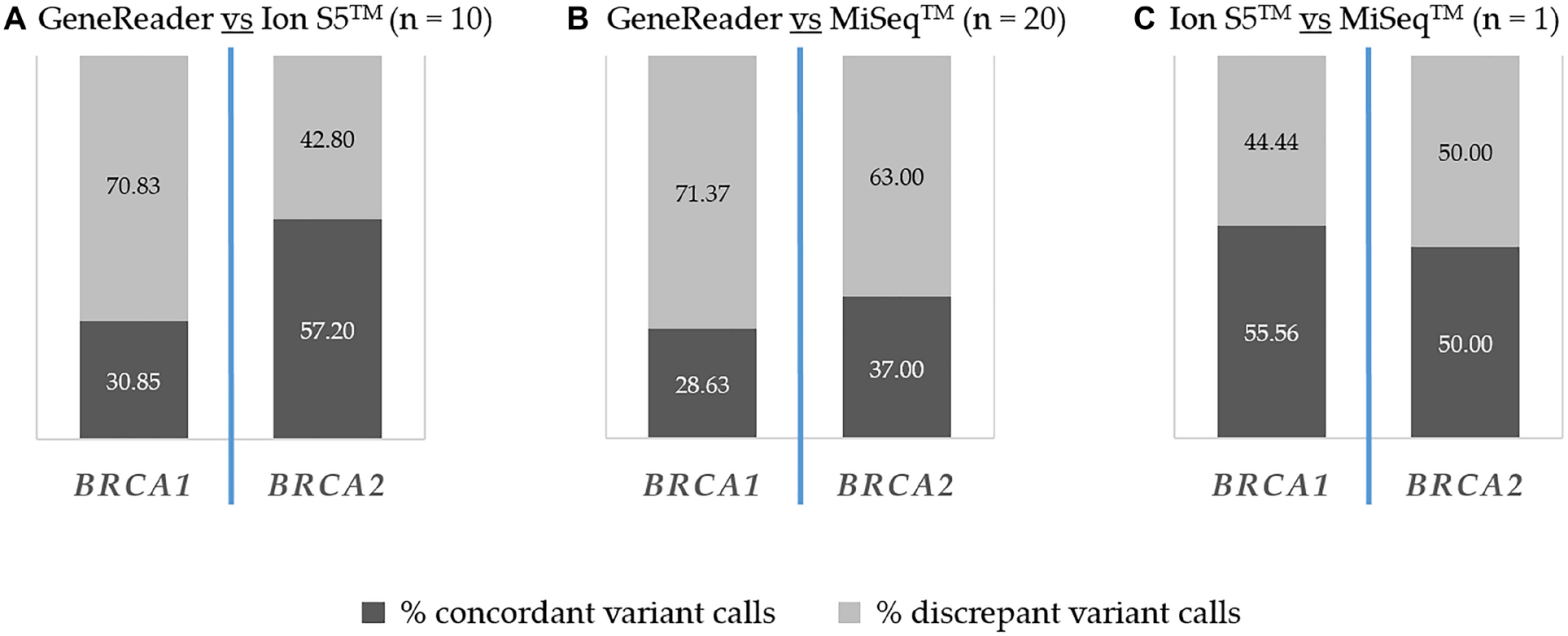
Inter-platform concordance of detected *BRCA1* and *BRCA2* alterations. Percentages of concordant (dark grey) and discrepant variant calls (light grey) are indicated. (**A**) GeneReader vs. Ion S5™. (**B**) GeneReader vs. MiSeq™. (**C**) Ion S5™ vs. MiSeq™.

As seen in Figure 2 the result was rather heterogeneous regarding all detected alterations, including benign and pathogenic ones as well as variants of unknown significance. In comparison, variants were assessed discrepant if they were detected by one assay but not the other, regardless of the degree of pathogenicity. The source of discrepancy was mainly coming from intron mutations in the comparison of GeneReader vs. MiSeq™ (*BRCA1:* 58%, *BRCA2*: 48%) as well as Ion S5™ vs. MiSeq™ (*BRCA1:* 100%, *BRCA2*: 80%). Differences in missense mutations were the most frequent in the comparison of GeneReader vs. Ion S5™ (*BRCA1:* 51%, *BRCA2*: 57%). Approximately 10% of the discrepant mutations in all comparisons and both genes were synonymous mutations and less than 5% were large indel mutations. The comparison was done on the basis of amino acid change.

Comparing GeneReader vs. Ion S5™ (Figure 2A), 30.85% and 57.2% of *BRCA1* and *BRCA2* mutations were detected with both instruments, while 70.83% *BRCA1* and 42.8% *BRCA2* mutations were detected either with the GeneReader or the Ion S5™.

In the comparison of GeneReader vs. MiSeq™ (Figure 2B), the proportions of concordant calls were 28.63% and 37%, and the proportions of discrepant calls were 71.37% and 63% for *BRCA1* and *BRCA2*, respectively. For the sample analyzed with both the Ion S5™ and the MiSeq™ (Figure 2C), 55.56% of the detected *BRCA1* variants were concordant, while 44.44% were discrepant. For *BRCA2*, half of the mutations were detected with both instruments or only with one.

## DISCUSSION

The aim of this study was to establish a uniform procedure for the detection and interpretation of *BRCA1* and *BRCA2* alterations in different *BRCA* associated tumor diseases and to facilitate routine diagnostics. 48 patient samples were sequenced with different NGS-based technology platforms. All samples were primarily analyzed with the GeneReader, and some samples were additionally analyzed with the Ion S5™ or the MiSeq™. In order to decide which device is most recommendable for individual requirements, various factors such as time, cost and quality of bioinformatics will be discussed in the following. A sufficient quality of data is an essential prerequisite to provide reliable and consistent results, as well as to achieve a valid, uniform *BRCA* testing. This includes the collection of relevant sample properties such as the tumor cell content and the DNA concentration inserted for the workflow, but also the quality of the individual sequencing runs, the panel-specific coverage as well as the correct annotation and classification of detected alterations. All these factors were taken into account in the assessment of the data collected in this study.

As described previously, all panels cover the exonic regions of *BRCA1* and *BRCA2* comparably well. Nevertheless, there are differences in the gene coverages of the respective panels. Compared to the QIAGEN panel, those from Thermo Fisher and Illumina^®^ also include larger intronic regions of the *BRCA* genes, enabling the detection of pathogenic alterations in exon/intron junction as well. Approximately 4% of all *BRCA1/2* alterations reported are splice site variants [26]. These may have a potential impact on pre-mRNA splicing, e.g., causing strong splicing defects like complete exon skipping or the activation of a cryptic donor site [27]. The disadvantage, however, is that considerably more benign or synonymous background mutations are detected, all of which have to be verified and interpreted. Especially in routine diagnostics this leads to an additional expenditure of time and effort. It would therefore be beneficial to establish helping tools that include a list of panel specific background mutations and artefacts that occur frequently. This list can be used as filter criteria for bioinformatic assessments. The results of the normal tissue samples analyzed in this study can also be helpful for this purpose, as it can be assumed that alterations in normal tissue have no clinical relevance or may have a germline consequence. Accordingly, more extensive panels like the Oncomine™ Comprehensive Assay v3 (Thermo Fisher) or Illumina’s AmpliSeq™ BRCA panel can be used, which enable the additional detection of clinically relevant alterations without increased effort.

Considering the genomic distribution of the detected *BRCA1* and *BRCA2* mutations, it was noticeable that exon 10 and exon 11 represented a “hotspot” region in both genes, respectively. Between 25% and 68% of the alterations have been found here. A possible explanation for the accumulation of point mutations in *BRCA1* exon 10 and *BRCA2* exon 11 could be the localization of RAD51 binding domains [27]. Mutations in this region could therefore influence or prevent the *BRCA* associated DNA damage response.

Considering the detected mutations with regard to their variant type, missense mutations (*BRCA1* 72%/*BRCA2* 61%) have been identified as the most common mutation type detected on the GeneReader and represented also large proportions (23–35%) on the other NGS platforms. One consequence of missense mutations is the translation of an altered protein with possible impairment of its function. In comparison, other researchers described frequencies of missense mutations of 39.83% for *BRCA1* and 45.91% for *BRCA2* [28]. Furthermore, intron mutations were more frequently detected with Thermo Fisher and Illumina^®^ than with QIAGEN, which might be due to the additional coverage of conserved intronic sequences. However, there were also platform-dependent differences. For example, 42% of the *BRCA1* alterations detected with the Ion S5™ were located in the intronic region, while the MiSeq™ detected even 60% of these mutations. A similar ratio was found for *BRCA2* (Ion S5™: 18%; MiSeq™: 35%). Numerous synonymous *BRCA1* (9–25%) and *BRCA2* (20–47%) variants were detected with all devices, which were mainly known polymorphisms and could be confirmed in some cases by the normal tissue samples analyzed, too.

The classification of the mutations detected with the GeneReader with respect to their pathogenicity was performed by QIAGEN’s bioinformatics software QCI-I. All detected alterations were then manually validated via the ClinVar database available online. Eight of 12 mutations were classified analogously as pathogenic. QIAGEN provided no interpretation for the alteration *BRCA2* c.3700_3704delGTAAA, classified as pathogenic in ClinVar and ARUP (*BRCA* Mutation Database, available at http://www.arup.utah.edu/), which was detected in sample 7. As this sample was normal tissue, a germline mutation can be assumed which has not been further investigated. The variant *BRCA2* c.1285_1286dupTT (sample 26) was classified as likely pathogenic by QCI-I, while *BRCA2* c.3544_3545delTT (sample 12) and *BRCA2* c.9097delA (sample 20) were classified as VUS. However, a comparison with ClinVar showed that all three mutations should be considered pathogenic. Since QCI-I also includes clinical studies and other annotations in its evaluation, it might be possible that one of these sources may have classified the respective mutation as likely pathogenic or VUS and adopted this result. Certainly, questionable is the fact that the *BRCA2* variant c.9097delA was classified as VUS in sample 20, as described above, but as pathogenic in sample 1. As the sequencing of sample 1 was performed in 2018, while sample 20 was sequenced in 2019, it is reasonable to conclude that in the meantime an update of the deposited sources has been carried out. Effectively, a classification as likely pathogenic has no clinical consequence, since these mutations were also reported in a molecular report. However, this would not necessarily be the case with variants of uncertain significance. Based on the measured quality parameters of the individual mutation as well as the clinical importance of the affected gene in the corresponding tumor entity, it is decided whether or not a mutation has been included in a final report. So, if an alteration is erroneously reported as VUS, it could have serious consequences for the patient. Especially, in the case of *BRCA* mutated breast and ovarian cancer. There are approved therapies from which a patient may not be able to benefit. Nevertheless, QIAGEN should check its bioinformatics software with regard to the mentioned inaccuracies, as they could be errors, which can have considerable consequences for patients.

Since the same samples of the study were analyzed with different NGS platforms, a comparison and thus a mutual internal validation of the results was possible. This showed that the *BRCA1* variant c.5467+2T>C could be detected with similar allele frequencies (AF) in both GeneReader (84.8%) and Ion S5™ (89.68%). Also, when comparing GeneReader versus MiSeq™, most pathogenic alterations were found with similar high AF. The only exception was the *BRCA2* variant c.9097delA in sample 20. The GeneReader detected this alteration with an AF of 8.79%, whereas the mutation on the MiSeq™ only showed an AF of 2.3% and should actually not be included in the analysis. However, since the mutation could be detected with both devices, it should be considered credible. In this context, it is also important to consider the tumor cell content of the respective sample of interest.

Another interesting pathogenic mutation was *BRCA2* c.8023A>G. In contrast to Ion S5™ and MiSeq™, this alteration was only found with the GeneReader in three samples (18, 20, 36) with AF <10%. A closer look at the analysis of the respective samples revealed that the mutations were only detectable in the reverse strand. The number of reverse reads with the detected variant were 13 (sample 18), 14 (sample 20) and 25 (sample 36), while the number of forward reads was 0 each time. The phenomenon known as strand bias describes the significant difference between the genotypes of forward and reverse strand [29]. Finally, this strand bias led to the low allele frequency of this mutation. Thus, the results must be evaluated critically in correlation with the histology and any previous reports in order to exclude artefacts.

If the clinical interest is directed only on the specific *BRCA* testing, Illumina’s AmpliSeq™ panel seems to be a good choice. With this panel, the exons of the two genes *BRCA1* and *BRCA2* were covered appropriately as well as exon/intron transitions. When comparing the three panels, AmpliSeq™ showed the largest coverage, which allowed a very accurate, disease-relevant diagnosis. However, a disadvantage was that more background mutations were detected. As Illumina’s analysis software BaseSpace only provides limited information on found variants and was additionally perceived rather confusing and less intuitively designed, it is helpful to be able to refer to a catalogue of known synonymous and benign mutations, such as the one compiled in this study.

In the context of mutational analysis of a *BRCA* associated tumor, not only the two *BRCA* genes are of interest, but also the so-called *BRCA*ness genes, which are mainly involved in DNA damage repair and cell cycle control. Therefore, for a more comprehensive diagnosis the Oncomine™ (OCA) panel is recommended. This panel covers 161 genes, including *BRCA1* and *BRCA2* as well as several *BRCA*ness genes involved in the repair mechanism, but also other genes, which are frequently altered in solid tumors and can contribute to tumorigenesis and tumor progression. The bioinformatics software Ion Reporter™ provides a general interpretation of all detected alterations as well as a gene specific analysis. Since the coverage of the two *BRCA* genes differed only marginally compared to the AmpliSeq™ panel, the OCA panel is an advisable alternative.

Looking at the QIAGEN panel from a coverage and evaluation perspective, it is inferior to the other NGS platforms in this study. The comparatively low genomic coverage of *BRCA1* and *BRCA2* is particularly noteworthy. This mainly includes the coding sequences of both genes. Thus, valid results could be obtained regarding the detection of pathogenic alterations in coding sequences, but beyond that, no statement could be made about mutations at splice sites or exon/intron transitions. For QIAGEN, the evaluation was performed with the software provided by the manufacturer and includes the analysis (QCI-A) as well as the interpretation (QCI-I) of the data. The software is clearly structured and user-friendly. Furthermore, the filter set can be changed and adapted manually. However, these were also features available within the bioinformatics software of Thermo Fisher and Illumina^®^.

In order to compare the workflows and bioinformatics of the three distinct devices, it is important to take factors such as handling, hands-on time and financial aspects into account, as well as clinical issues, e.g. required sensitivity or genes of interest. In regard to handling, QIAGEN is currently working on the automation of its workflows. The whole QIAGEN equipment involved in sample processing and library preparation has been designed to allow for automated performance. However, automation is not available yet, so that the hands-on time is considerably longer than with Thermo Fisher or Illumina^®^. In addition, the QIAGEN workflow requires not only the GeneReader but also other company equipment (e.g., QIACube^®^ and QIAxcel^®^) in order to perform a complete sequencing run. With Thermo Fisher, clonal amplification can be performed automatically. For this purpose, the Ion Chef™ is required as an additional instrument. The Ion Chef™ is able to work overnight and loads a maximum of two ion semiconductor chips with the libraries to be sequenced. With Illumina^®^ no other equipment from the manufacturer is required apart from the MiSeq™. Furthermore, a simple thermal cycler from any manufacturer is necessary for all workflows as well as a device for the determination of DNA quality, e.g. Qubit or real-time PCR. The reduced hands-on time simplifies the handling of Thermo Fisher and Illumina^®^ workflows.

An advantage for routine diagnostics is that all protocols include safe stopping points where the processed samples can be stored for hours, weeks or even months. This means that all diagnostic methods performed in a laboratory can be paused or continued as required. However, it should be noted that the processing time from case acceptance to final report recommended in the guideline should not exceed 10 working days. Especially in the interest of patients, a fast analysis should be achieved, since in case of doubt, decisions on therapies that are essential for survival may depend on it. For sequencing faster Thermo Fisher has launched a new automated system, known as Genexus.

Finally, there are also economic and financial differences between the individual NGS platforms. The costs per case increase according to the number of genes included in the panel. Thus, the OCA panel is comparatively more cost-intensive, but may also offer additional therapy options for the patient.

However, it should be noted that all manufacturers label their manuals “For Research Use Only. Not for use in diagnostic procedures.” and thus make themselves unassailable. Each laboratory must therefore establish and validate the selected NGS method internally in order to identify limits and pitfalls also with regard to bioinformatics evaluation under the aspect of maximum patient care. For quality assurance, participation in laboratory ring trials is also advisable. Each lab should perform a validation of the whole NGS workflow including the final annotation.

## MATERIALS AND METHODS

### Patient collective

The study included 48 patients. The cohort’s mean age (range) was 58 (30–83) years, with most patients (47.92%) aged between 60 and 79 years; 79.17% were female. Tumor entities with an increased risk of disease due to *BRCA1/2* mutations were implicated: 21 patients (43.75%) harbored a tumor of the ovaries. Other gynecological tumors were breast cancer (2) as well as carcinomas of the fallopian tube (2), uterus (6) and cervix (4). Eight patients carried a prostate and two a pancreatic carcinoma. In addition to the 45 tumor samples, normal tissue of three patients with breast cancer was also included. The study compared the results of three different NGS platforms. All samples were primarily sequenced with the GeneReader (QIAGEN). Additionally, 10 tumor samples were sequenced with the Ion S5™ (Thermo Fisher) as well as 19 tumor samples and one normal tissue sample with the MiSeq™ (Illumina^®^).

### Library preparation

All laboratory work was performed according to the respective manufacturer’s protocol available online. The preparation of formalin-fixed, paraffin-embedded (FFPE) tissue samples was performed identically for all three workflows. The purification of DNA from the FFPE tissue samples was performed using the Maxwell^®^ RSC Instrument (Promega Corporation) with the Maxwell^®^ RSC FFPE Plus DNA Kit (Promega Corporation). Therefore, the tumor containing area of a tissue section or respectively the total area of a normal tissue section was scraped off the slide, placed into a 1.5 ml tube and centrifuged at maximum speed for 15 sec. 20 μl of 20 mg/ml Proteinase K and 180 μl Incubation Buffer was added. Overnight samples were heated at 70°C. After incubation the samples were mixed with 400 μl Lysis Buffer and transferred to the Maxwell^®^ RSC Cartridge. Further preparation and the instrument run were performed according to manufacturer’s protocol. The concentration of DNA was measured with the Qubit 4 Fluorometer (Invitrogen). For the library preparation, panel-specific amounts of DNA were used as input: 40 ng for the GeneRead™ QIAact BRCA UMI Panel (QIAGEN), 20 ng for the Oncomine™ Comprehensive Assay v3 (Thermo Fisher) and 20 ng for the AmpliSeq™ BRCA Panel (Illumina^®^).

### Next-generation sequencing

Data analysis was performed using the analysis software platforms provided by the respective manufacturers. QIAGEN: QIAGEN Clinical Insight Analyze (QCI-A) for GeneReader 1.5.0 and QIAGEN Clinical Insight Interpret (QCI-I). Thermo Fisher: The primary analysis of the sequencing data was completed by Torrent Suite™ software. Afterwards data were analyzed with the Ion Reporter™ software (version 5.12.0.0), filter chains Oncomine Variants 5.10 and Oncomine Extended 5.12 were used. Illumina: BaseSpace Variant Interpreter, version 2.9.1.15.

Each platform determines different quality parameters and corresponding cut-off values. In all three groups there were tumor samples that showed restricted quality in a few of these quality parameters (QIAGEN: 40%, Thermo Fisher: 20%; Illumina: 15%). However, since most parameters were fulfilled sufficiently, these samples were still included in the analysis.

### Data analysis

Genomic alterations were identified by the alignment on the reference genome hg19 (GRCh37) available at https://www.ncbi.nlm.nih.gov/. To achieve reliable results, only alterations with fulfilled quality criteria were considered, such as allele frequency ≥5% and a coverage of at least 100× for the GeneReader and 500× for the Ion S5™ and the MiSeq™. Classification and interpretation of detected filtered and unfiltered variants of *BRCA1/2* were evaluated. The variant annotation provided by the respective software was manually reviewed according to the online databases ClinVar [30] and Cosmic [31]. Other databases used for validation were: ARUP, BRCA Exchange, gnomAD [32], OncoKB, dbSNP and cBioPortal (available online). For this study, the annotation of pathogenicity of the detected variants was determined according to the ClinVar classification in: “benign”, “likely benign”, “uncertain significance”, “likely pathogenic”, “pathogenic”. To achieve a consistent approach of naming all variants, sequence variant nomenclature was carried out in concordance with the guidelines by the Human Genome Variation Society (HGVS) [33].

## CONCLUSIONS

**Figure 3:**
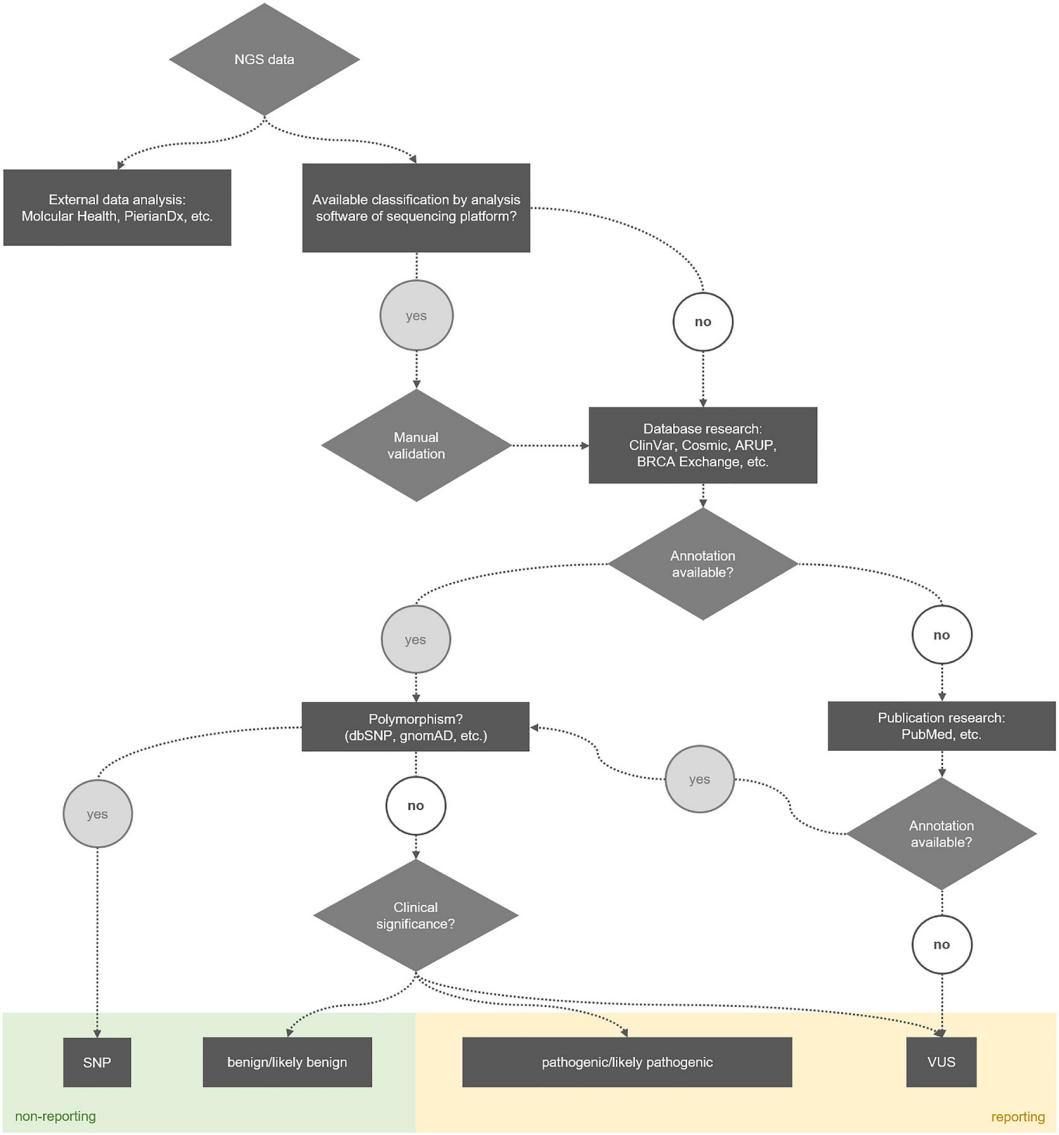
Process for identifying the clinical significance of alterations detected with NGS.

In summary, the three distinct NGS platforms used in this study detected a varying number of alterations. Both false-negative and false-positive detected alterations can have serious consequences for patients. On the one hand, a patient with a false-negative result may not receive life-saving therapy. On the other hand, a patient with a false-positive result may be treated but suffers from the side effects of treatment and does not have any clinical benefit in the end. The tumor cell content plays an important role as well. In case it is rather low, it is possible that even with a well-covered pathogenic alteration, most cells in the tissue would not respond to therapy. Furthermore, some mutations were annotated differently depending on the NGS platform, which could also lead to an altered clinical consequence. Currently, a manual check of the detected variants is required, independent of the respective NGS platform and software used. Within the scope of this study, supporting tools were created to simplify the handling of generated NGS data in the context of reporting. On the one hand, an overview of benign *BRCA1/2* alterations and polymorphisms, which are frequently found in the population (Table 3). This allows the focus of the evaluation to be shifted to pathogenic, therapy-relevant mutations or the closer examination of VUS. On the other hand, the process of identifying the clinical significance of detected alterations was illustrated in a flow chart (Figure 3), including the relevant databases and the decision whether or not to integrate an alteration into the final report. Currently, all detected alterations are evaluated according to this process, but other factors such as fulfilled quality criteria (allele frequency, coverage), tumour cell content as well as the DNA concentration of the starting material are finally decisive for the reporting. With the help of the newly gained knowledge, the analysis can be performed much more effectively and efficiently despite a plethora of sequencing results.

Although the OCA panel is the most expensive per case, it can be recommended from this study being most appropriate for current and prospective requirements of molecular analysis. The annotations of the Ion Reporter™ software are consistent with known databases, so that a reliable report can be generated. In addition, the broad number of genes covered by the OCA panel may open up new treatment options for patients. Molecular profiling is particularly important for patients with *BRCA1/2* mutations, as there are effective approved therapies. Due to the large protein complex in which the BRCA proteins function, mutations in other *BRCA*ness genes, which are involved in DNA repair including homologous recombination, may also be crucial, as these are also associated with a response to PARP inhibitors. Currently, distinct systems including Oncoscan arrays are tested to assess distinct deficiencies in homologous repair mechanism.

A new possibility to process molecular information more easily is to pass on the raw NGS data to a service provider such as Molecular Health [34] or PierianDx [35]. These companies offer tertiary analyses through a combination of artificial intelligence and machine learning and can convert large amounts of data into precise reports. Molecular and clinical data of individual patients are assessed in comparison to global medical, biological and pharmacological knowledge in order to make more accurate decisions regarding diagnosis, therapy and drug safety.
