# Addendum: Sequencing of *BRCA1/2*-alterations using NGS-based technology: annotation as a challenge

**DOI:** 10.18632/oncotarget.28483

**Published:** 2023-08-30

**Authors:** Silvana Ebner, Ria Winkelmann, Saskia Martin, Jens Köllermann, Peter J. Wild, Melanie Demes

**Affiliations:** ^1^Dr. Senckenberg Institute of Pathology, University Hospital Frankfurt, Frankfurt am Main, Germany; ^2^Wildlab, University Hospital MVZ GmbH, Frankfurt am Main, Germany

**This article has an addendum:** No external funding was obtained.

Original article: Oncotarget. 2022; 13:464–475. 464-475. https://doi.org/10.18632/oncotarget.28213

